# An evaluation of the preprints produced at the beginning of the 2022 mpox public health emergency

**DOI:** 10.1186/s41073-024-00152-w

**Published:** 2024-10-07

**Authors:** Melanie Sterian, Anmol Samra, Kusala Pussegoda, Tricia Corrin, Mavra Qamar, Austyn Baumeister, Izza Israr, Lisa Waddell

**Affiliations:** 1 Public Health Risk Sciences Division, National Microbiology Laboratory, Public Health Agency of Canada, Guelph, Canada; 2 Department of Population Medicine, University of Guelph, Guelph, Canada

**Keywords:** Mpox, Monkeypox, Preprint, Peer-review, Quality, Risk of bias

## Abstract

**Background:**

Preprints are scientific articles that have not undergone the peer-review process. They allow the latest evidence to be rapidly shared, however it is unclear whether they can be confidently used for decision-making during a public health emergency. This study aimed to compare the data and quality of preprints released during the first four months of the 2022 mpox outbreak to their published versions.

**Methods:**

Eligible preprints (*n* = 76) posted between May to August 2022 were identified through an established mpox literature database and followed to July 2024 for changes in publication status. Quality of preprints and published studies was assessed by two independent reviewers to evaluate changes in quality, using validated tools that were available for the study design (*n* = 33). Tools included the Newcastle-Ottawa Scale; Quality Assessment of Diagnostic Accuracy Studies 2 (QUADAS-2); and JBI Critical Appraisal Checklists. The questions in each tool led to an overall quality assessment of high quality (no concerns with study design, conduct, and/or analysis), moderate quality (minor concerns) or low quality (several concerns). Changes in data (e.g. methods, outcomes, results) for preprint-published pairs (*n* = 60) were assessed by one reviewer and verified by a second.

**Results:**

Preprints and published versions that could be evaluated for quality (*n* = 25 pairs) were mostly assessed as low quality. Minimal to no change in quality from preprint to published was identified: all observational studies (10/10), most case series (6/7) and all surveillance data analyses (3/3) had no change in overall quality, while some diagnostic test accuracy studies (3/5) improved or worsened their quality assessment scores. Among all pairs (*n* = 60), outcomes were often added in the published version (58%) and less commonly removed (18%). Numerical results changed from preprint to published in 53% of studies, however most of these studies (22/32) had changes that were minor and did not impact main conclusions of the study.

**Conclusions:**

This study suggests the minimal changes in quality, results and main conclusions from preprint to published versions supports the use of preprints, and the use of the same critical evaluation tools on preprints as applied to published studies, in decision-making during a public health emergency.

**Supplementary Information:**

The online version contains supplementary material available at 10.1186/s41073-024-00152-w.

## Background

The scientific peer-review process can take three to four months for many medicine and public health-related journals [1]. Preprints are scientific manuscripts that are publicly accessible prior to undergoing the formal peer-review process [2]. They allow for rapid dissemination of new research to the scientific community and general public. The utility of preprints was highlighted during the COVID-19 pandemic, when the number of preprint articles being posted rapidly increased, providing researchers with timely access to the most up-to-date evidence for public health response activities [3].

A major concern of using preprints for evidence-based decision-making is their potentially poor quality and credibility due to no formal peer-review process. Therefore, it is important to determine whether preprints can be relied upon as sources of new scientific evidence, instead of solely peer-reviewed literature, by comparing the quality and data of preprints to their published counterparts. Previous research has examined discrepancies between COVID-19 preprints and their subsequent journal publications, such as changes in outcomes, numerical results, methods, main conclusions in the abstract, and general reporting characteristics [4–6]. However, this prior research has been limited to comparing abstracts or specific study designs.

An mpox evidence surveillance database created at the onset of the 2022 mpox (previously known as monkeypox) outbreak [7] allowed for comparison of preprints and their subsequent published versions, across a range of study designs. The objective of this study was to evaluate the utility of preprints for decision-making during a public health emergency, by comparing the data and quality of preprints released in the first four months of the 2022 mpox outbreak with their published counterparts, as well as the quality between unpublished and published preprints.

## Methods

A protocol was developed a priori for this study, which includes the search strategy, eligibility criteria, quality assessment tools, and the data characterization form (Additional file 1). The protocol was made available on Open Science Framework after the research concluded (10.17605/OSF.IO/D3V9K). There were a few small deviations from the original protocol made after piloting the tools, these are noted in Additional file 1.

### Research questions

The following research questions were used for this investigation:

1. Among the preprints that were posted between May to August 2022 and published by July 2024, do potential changes in quality or data between the preprint and published version impact the main conclusions?
2. Among the preprints that were posted between May to August 2022 and published by July 2024, are there differences in quality between preprints that were published compared to those that were not?

### Information sources and search strategy

Mpox evidence surveillance was conducted from May 2022 onward and included a comprehensive search strategy developed and tested through an iterative process by an experienced information specialist in consultation with the review team and peer-reviewed by international colleagues [7]. PubMed, Scopus, EuropePMC, SSRN, and arXiv were searched twice weekly between May 1, 2022 to December 31, 2022 and then weekly until June 2023 to identify preprints and published literature on mpox. The searches were adapted to each database and utilized keywords such as monkeypox, mpox, simianpox, MPXV, variole du singe, and variole simienne. There were no restrictions on language; however, the search was constructed using English and French terms for mpox. Since May 2022, results of all primary and non-primary literature were maintained in RefWorks [8], DistillerSR [9] and a searchable Excel database, referred to as the mpox database herein. Detailed methods used to generate and update the mpox database are described elsewhere [7]. Each citation in the mpox database was categorized according to literature type (primary or non-primary), study design, and publication status (preprint or published). Categorization was performed by one reviewer and spot-checked by a senior reviewer. For this study, eligible mpox preprints were identified by filtering these categories.

### Eligibility criteria and selection process

The first version of all primary research studies posted as preprints between May 1 and August 22, 2022 in English and French were included. August 22 was selected as the end-date as this was when the incidence of global mpox cases was consistently declining [10]. Preprints were followed through to July 2024 for publication status, which was 23 to 26 months after the preprint was first released. Publication status was verified through indications of publication directly on the preprint article page or manually searching on Google or Google Scholar. Preprints and their published versions were then linked in the mpox database.

Non-primary (e.g. reviews) and methods studies were excluded because these studies did not provide primary results that could be compared between a preprint and published version. Only the first version of each preprint was assessed as it was the first available for use by decision-makers, and subsequent versions may have undergone some peer-review and/or changes may have been made to results.

### Quality assessment

While data was extracted for all identified study designs, quality was assessed using validated quality assessment tools for study designs that had an applicable tool: Newcastle-Ottawa Scale (NOS) for case-control and cohort studies [11]; the adapted version of the NOS for cross-sectional studies [12, 13]; Quality Assessment of Diagnostic Accuracy Studies 2 (QUADAS-2) [14]; JBI Critical Appraisal Checklist for case series [15]; and JBI Critical Appraisal Checklist for prevalence studies [16]. The latter tool was adapted for surveillance data analyses [16] by removing one criteria assessing adequate sample size as it was not applicable to this study design. Each tool guides the reviewer through a set of questions to assess whether the paper is high quality (no concerns with study design, conduct, and/or analysis), moderate quality (minor concerns) or low quality (several concerns). A description of each tool is provided in the protocol (Additional file 1: Appendix 3).

Quality assessment was performed independently in duplicate using DistillerSR, a web-based systematic review management program [9]. For each quality assessment tool, two studies (preprint or published) were piloted by the two assigned reviewers to ensure consistency and clarity in using the tool. Conflicts were resolved by consensus or consultation with a third reviewer if consensus could not be reached. For preprint-published pairs, quality assessment was completed separately by the same reviewers: one for the preprint and one for the published version. Quality was also assessed for unpublished preprints where a validated quality assessment tool was available.

### Data characterization and utility

Data characterization was performed only for studies that had both a preprint and published version. Prepopulated data from the mpox database comprised of citation information, study design, and preprint posting date. The data characterization and utility form captured changes in general reporting characteristics (e.g., author list, funding, conflicts of interest), changes in the abstract, changes in methods (e.g., sample size, study period, statistical analysis, other), changes in outcomes, and changes in results along with their impact on main conclusions (Additional file 1).

The form was piloted by all reviewers on a random sample of five preprint-published pairs of different study designs and adjusted as needed. Data characterization was performed in DistillerSR using an accelerated process of reviewing, in which a senior reviewer (AB, KP, LW, TC) verified the extraction form completed by a junior reviewer (AS, II, MQ, MS). Reviewers examined the preprint and published version concurrently and completed one data characterization form for each pair. Conflicts were resolved by consensus or consultation with a third reviewer if consensus could not be reached.

### Data synthesis

The datasets for quality assessment and data extraction were exported from DistillerSR into Microsoft^®^ Excel^®^ Version 2311 (Additional file 2 and 3). Quality assessment results were categorized and tabulated according to study design. Changes in data were summarized using descriptive statistics in Excel, and tables were used to display summarized changes in methods and results. Results were narratively synthesized.

## Results

### Characteristics of the included studies

Ninety-four preprints posted between May 1 to August 22, 2022 were identified from the mpox database. Methods studies (*n* = 7) and non-primary studies (*n* = 11) were excluded, resulting in 76 relevant preprints included. Among these, 60 were published by July 24, 2024, and 16 remained unpublished. Data extraction was conducted for the 60 preprint-published pairs (Additional file 2).

There were 14 different study designs across the included studies (Table 1). All of the included cross-sectional studies, case series, in vitro studies, predictive models, exposure investigations, and cluster investigations were published by July 2024, as well as most of the mathematical models, surveillance data analyses, and in silico studies. In comparison, only half of the phylogenetic analyses and diagnostic test accuracy studies were published (Table 1). Quality assessment was conducted for 33 studies (Additional file 3).

**Table 1:** Number of published and unpublished preprints, categorized by study design and corresponding quality assessment tool

Study Design	Quality Assessment Tool	% Published	Published Preprints	Unpublished Preprints
**Modelling studies**				
Predictive model	None available	100%	9	0
Mathematical model	None available	75%	6	2
**Observational studies**				
Cross-sectional	NOS^a^	100%	7	0
Cohort	NOS^a^	67%	2	1
Case control	NOS^a^	100%	1	0
Surveillance data analysis	JBI Critical Appraisal Checklist for prevalence studies	75%	3	1
**Descriptive studies**				
Case series	JBI Critical Appraisal Checklist for case series	100%	7	0
Cluster investigation	None available	100%	1	0
Exposure investigation	None available	100%	3	0
**Other**				
In vitro	None available	100%	6	0
In silico	None available	67%	4	2
Phylogenetic analysis	None available	50%	5	5
Diagnostic test accuracy	QUADAS-2^a^	55%	6^b^	5
**Total**		**79%**	**60**	**16**

^a^*NOS* Newcastle-Ottawa Scale, *QUADAS-2* Quality Assessment of Diagnostic Accuracy Studies 2

^b^For one study the preprint was classified as a bioinformatic analysis since it presented preliminary analysis of potential antigenic targets whereas the published version fit the criteria for a diagnostic test accuracy study and was classified as such. Only the published version underwent quality assessment

### Changes in quality

Twenty-five preprint-published pairs and seven unpublished preprints underwent quality assessment; for one additional study, only the published version underwent quality assessment since it provided diagnostic test accuracy data while the preprint only provided a bioinformatic analysis.

### Case-control, cohort and cross-sectional studies

The NOS is composed of three domains that assess risk of bias: selection of the study groups, comparability of the groups, and ascertainment of the outcome. Different versions of the NOS were used to evaluate cohort, case-control, and cross-sectional studies (Table 2) [11–13]. The NOS produces a total quality score for each study, which is the sum of points earned across the three domains. A study that increased in total quality score from preprint to published could still have low overall quality if there remained several concerns with study design, conduct and/or analysis.

There was one case-control study assessed for quality, which was a preprint-published pair. The total quality score remained the same between the preprint and published version [17, 18].

There were seven cross-sectional studies assessed for quality, all of which were preprint-published pairs. For four out of seven pairs, the total quality score was higher by one to two points in the published version compared to the preprint, because the published version reported the sample size (Selection domain) or reported controlling for age, sex, and other factors (Comparability domain) [19–26]. For one pair, the total quality score was lower by one point in the published version compared to the preprint, because the published version did not report the statistical test used (Outcome domain) [27, 28]. Despite the one to two point variation in the total score for these five pairs, the overall quality was low for both preprint and published versions.

There were three cohort studies assessed for quality, which included two preprint-published pairs and one unpublished preprint. For one out of two pairs, the total quality score was higher by one point for the published version compared to the preprint, as only the published version clearly reported how the presence of symptoms were assessed at follow-up (Outcome domain) [30, 31]. However, the overall quality was still considered low for both the preprint and published version. There were no clear differences in quality between the one unpublished cohort preprint [32, 33] and the two published cohort preprints.

**Table 2:** Quality assessment of cross-sectional, cohort, and case-control studies using the Newcastle-Ottawa Scale

Study design	Study	Publication status	Selection (/3 or /4)	Comparability (/2)	Outcome^a^ (/2 or /3)	Total score (/7 or /9)	Risk of bias^b^
Case-control	Yinka-Ogunleye [17]	Preprint	3/4	2	2/3	7/9	Medium
		Published	3/4	2	2/3	7/9	Medium
Cross-sectional	Ahmed [19, 20]	Preprint	1/3	**0**	2/2	3/7	High
		Published	1/3	**2**	2/2	5/7	High
	Aljamaan [21, 22]	Preprint	**0/3**	0	2/2	2/7	High
		Published	**1/3**	0	2/2	3/7	High
	Alshahrani [23, 24]	Preprint	1/3	**0**	2/2	3/7	High
		Published	1/3	**2**	2/2	5/7	High
	Malik/ Winters [25, 26]	Preprint	**0/3**	2	2/2	4/7	High
		Published	**1/3**	2	2/2	5/7	High
	Temsah [34, 35]	Preprint	0/3	0	2/2	2/7	High
		Published	0/3	0	2/2	2/7	High
	Wang [27, 28]	Preprint	0/3	2	**2/2**	4/7	High
		Published	0/3	2	**1/2**	3/7	High
	Wang [29, 36]	Preprint	0/3	2	2/2	4/7	High
		Published	0/3	2	2/2	4/7	High
Cohort	De Baetselier [30, 31]	Preprint	2/4	0	**2/3**	4/9	High
		Published	2/4	0	**3/3**	5/9	High
	Arbel [32]	Unpublished preprint	3/4	2	1/3	6/9	Medium
	Zucker [33, 37]	Preprint	3/4	2	3/3	8/9	Low
		Published	3/4	2	3/3	8/9	Low

^a^For case-control studies, exposure was evaluated instead of outcome (according to the NOS for case-control)

^b^Bolding indicates change in score for the domain

### Case series

All seven case series were preprint-published pairs and evaluated using the JBI Critical Appraisal Checklist for Case Series, which consists of 10 quality criteria (Table 3) [38–51]. One study met an additional criterion in the published version because it provided sufficient information that mpox was measured in a standard, reliable way for all participants (criteria two) whereas the preprint did not [42, 43]. Overall, most criteria in the checklist were met across the studies, and the criteria addressed between preprint and published versions remained similar, suggesting no change in quality.

**Table 3:** Critical appraisal of case series using the JBI Critical Appraisal Checklist for Case Series

Study	Publication status	Criteria addressed^a^									
		Inclusion criteria	Uniformly applied outcome measure	Outcome measurement	Consecutive inclusion	Complete inclusion	Demographics	Clinical information	Outcomes and follow up results	Clinic information	Appropriate statistics
Girometti [38, 39]	Preprint	Yes	Yes	Yes	Yes	Yes	Yes	Yes	Yes	Yes	NA
	Published	Yes	Yes	Yes	Yes	Yes	Yes	Yes	Yes	Yes	NA
Noe [40, 41]	Preprint	No	Yes	Yes	Yes	No	Yes	Yes	Yes	Yes	NA
	Published	No	Yes	Yes	Yes	No	Yes	Yes	Yes	Yes	NA
Patalon [42, 43]	Preprint	No	**No**	Yes	No	No	Yes	Yes	Yes	No	NA
	Published	No	**Yes**	Yes	No	No	Yes	Yes	Yes	No	NA
Pittman [44, 45]	Preprint	Yes	Yes	Yes	Unclear	Unclear	Yes	Yes	Yes	Yes	Yes
	Published	Yes	Yes	Yes	Unclear	Unclear	Yes	Yes	Yes	Yes	Yes
Tarín-Vicente [46, 47]	Preprint	Yes	Yes	Yes	Yes	Yes	Yes	Yes	Yes	Yes	Yes
	Published	Yes	Yes	Yes	Yes	Yes	Yes	Yes	Yes	Yes	Yes
Thy [48, 49]	Preprint	Yes	Yes	Yes	Yes	Yes	Yes	Yes	Yes	Yes	NA
	Published	Yes	Yes	Yes	Yes	Yes	Yes	Yes	Yes	Yes	NA
Yadav [50, 51]	Preprint	No	Yes	Yes	Yes	No	Yes	Yes	Yes	Yes	NA
	Published	No	Yes	Yes	Yes	No	Yes	Yes	Yes	Yes	NA

^a^Yes indicates criteria met, no indicates criteria not met, unclear indicates lack of reporting. NA refers to criteria that are not applicable for that study. Bolding indicates change in reporting or quality for particular criteria

### Surveillance data analyses

Three preprint-published pairs were evaluated using an adapted version of the JBI Critical Appraisal Checklist for Prevalence Studies, which used eight of the nine criteria that assess quality (Table 4) [52–58]. These surveillance data analyses lacked in reporting several criteria on the JBI Critical Appraisal Checklist. There were no improvements between the preprint and published versions, and no differences between the three published and one unpublished preprint (Table 4) [52–58].

**Table 4:** Critical appraisal of surveillance data analyses using the JBI Critical Appraisal Checklist for Prevalence Studies

Study	Publication status	Criteria addressed^a^							
		Sampling frame	Sampling strategy	Sample description	Sufficient coverage of subgroups	Outcome measurement	Uniformly applied outcome measure	Appropriate statistics	Response rate
de Jonge [53, 54]	Preprint	Yes	Yes	Yes	Unclear	Yes	Yes	NA	NA
	Published	Yes	Yes	Yes	Unclear	Yes	Yes	NA	NA
Miura [55, 56]	Preprint	Yes	Yes	No	Unclear	Yes	Yes	Yes	Unclear
	Published	Yes	Yes	No	Unclear	Yes	Yes	Yes	Unclear
Wurtzer [57, 58]	Preprint	Yes	Unclear	No	Unclear	Yes	Yes	NA	NA
	Published	Yes	Unclear	No	Unclear	Yes	Yes	NA	NA
Charniga [52]	Unpublished	Yes	Yes	No	Unclear	Yes	Yes	Yes	Unclear

^a^Yes indicates criteria met, no indicates criteria not met, unclear indicates lack of reporting. NA indicates that the criteria was not applicable for that study

### Diagnostic test accuracy studies

Five preprint-published pairs and five unpublished preprints were evaluated using the QUADAS-2 tool, and for one study only the published version was evaluated, since the preprint included only a preliminary bioinformatic analysis [59–70]. The QUADAS-2 tool assesses whether the following four domains introduce bias: selection of patients, conduct or interpretation of the index test, the reference standard (including its conduct or interpretation), and patient flow and timing (e.g. appropriate intervals between index test and reference standard, all patients included in analysis) [14]. Three out of five preprint-published pairs had changes in at least one domain (Table 5). Both the unpublished and published preprints had several domains rated as unclear or high ROB, and some lacked reporting on applicability concerns; overall there was no distinguishing pattern in quality between these two groups.

**Table 5:** Risk of bias and applicability assessment of diagnostic test accuracy studies using the QUADAS-2 tool

		Risk of bias^a^				Applicability concerns		
Study	Publication status	Patient selection	Index test	Reference standard	Flow and timing	Patient selection	Index test	Reference standard
Albin [71, 72]	Preprint	Low	Low	Low	Low	Low	Low	Low
	Published	Low	Low	Low	Low	Low	Low	Low
Allan-Blitz [60, 61]	Preprint	**Unclear**	Unclear	Unclear	Unclear	**Unclear**	Low	Low
	Published	**Low**	Unclear	Unclear	Unclear	**Low**	Low	Low
La Rosa [64, 65]	Preprint	Low	Low	Low	Low	Low	Low	Low
	Published	Low	Low	Low	Low	Low	Low	Low
Nörz [73, 74]	Preprint	**High**	**Unclear**	Unclear	Low	Low	Low	**Low**
	Published	**Unclear**	**Low**	Unclear	Low	Low	Low	**Unclear**
Wang [66, 67]	Preprint	**High**	Unclear	Low	Low	Low	Low	Low
	Published	**Unclear**	Unclear	Low	Low	Low	Low	Low
Wu^b^ [70]	Published	High	Unclear	Unclear	Low	High	Unclear	Unclear
Ali [59]	Unpublished	High	Low	Unclear	Unclear	Unclear	Low	Unclear
Bhadra [62]	Unpublished	Low	Unclear	Low	Low	High	Low	High
Islam [63]	Unpublished	High	Unclear	Low	Unclear	Unclear	Unclear	Low
Wawina-Bokalanga [68]	Unpublished	Low	Unclear	Unclear	Low	Low	Low	Low
Wolfe [69]	Unpublished	Low	Low	Low	Low	Low	Low	Low

^a^Low indicates low risk of bias, high indicates high risk of bias, and unclear indicates lack of reporting. Bolding indicates a change in quality for that domain

^b^Only the published version was classified as a diagnostic test accuracy study and evaluated for quality; the preprint was classified as a bioinformatic analysis [62]

### Changes in data between preprints and their published versions

Changes in general reporting characteristics, abstracts, methods, outcomes, and results were examined for 60 preprint-published pairs, hereafter referred to as “studies”.

#### General reporting characteristics

Of the general reporting characteristics evaluated, authorship changed in 23% (14/60) of studies (Additional file 4: Supplementary Table 1). Almost all changes in authorship involved adding authors, with a median of two (range: 1–10) authors added. The funding statement changed in 28% (17/60) of studies and mainly involved adding funding sources or more detailed information on sources. The funding sources added were either public/governmental or academic institutions (e.g. European Union, National Institutes of Health, Berlin University Alliance). In 15% (9/60) of studies, only the preprint reported the funding statement. For one study only the published version reported funding [75, 76], and for two studies funding was absent from both versions [77–80]. The conflict of interest statement changed in 13% (8/60) of studies. Almost all changes to the conflict of interest statement involved adding conflicts in the published version; among the seven studies with added conflicts, six were related to pharmaceutical or biotechnology companies [38, 39, 48, 49, 81–88]. In 4% (2/53) of preprint-published pairs, the conflict of interest statement was only included in the preprint version. Overall, the only concerning change in general reporting characteristics was the addition of conflicts of interest in the published versions.

#### Abstracts

The authors highlighted different results in the abstract of the published version for 42% (25/60) of studies, while 45% (27/60) had no changes. 13% (8/60) of studies provided an abstract in the preprint but did not provide an abstract in the published version; all of these were published as an article type that typically does not include an abstract, such as a correspondence or letter to the editor. Among the 25 studies that highlighted different results, results tended to be added (56%; 14/25) rather than removed (20%; 5/25) in the published version. A change within one or more results (e.g. numerical result changed) occurred in 40% (10/25) of studies, and details in results (e.g. p-values) were removed from the published version in 15% (3/20).

#### Methods

**Fig. 1:**
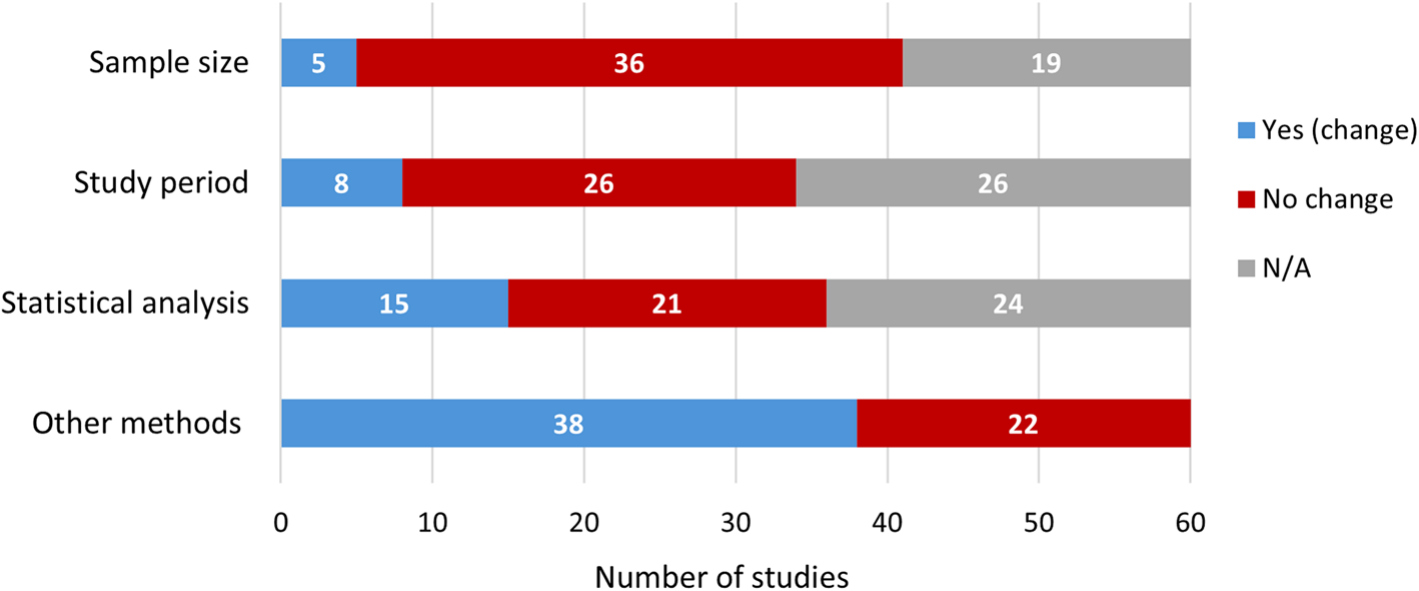
Changes in methods from the preprint to published version (*N* = 60). N/A refers to studies that do not report that particular method in either the preprint, published version, or both versions

73% (44/60) of studies had a change in at least one aspect of their methodology from the preprint to published version (Fig. 1). Among the 41 studies that reported a sample size, 12% (5/41) underwent a change, of which three had a larger sample size [57, 58, 89–92] and two had a smaller sample size in the published version [33, 37, 73, 74]. Of the 34 studies that reported a study period, 24% (8/34) had longer (*n* = 6) [33, 37, 57, 58, 70, 89–95], shorter (*n* = 1) [46, 47], or more specific (*n* = 1) [96, 97] study periods in the published version. Among the 36 studies that reported statistical analyses, 42% (15/36) had changes in the published version. In 63% (38/60) of studies, there were also changes in “other methods”, which encompassed a range of changes such as reporting additional methods pertaining to added outcomes (*n* = 10) [30, 31, 57, 58, 60, 61, 70, 75, 76, 91–93, 98–105], additional details (*n* = 11) [21, 22, 30, 31, 57, 58, 60, 61, 79, 80, 89, 90, 96, 97, 106–113], a higher number of sequences used in analysis (*n* = 5) [70, 75, 76, 93, 98, 99, 114–117], adjustments to model assumptions (*n* = 3) [85, 86, 118–122], and reporting of questionnaires (*n* = 2) [25, 26, 34, 35].

#### Outcomes and results

**Fig. 2:**
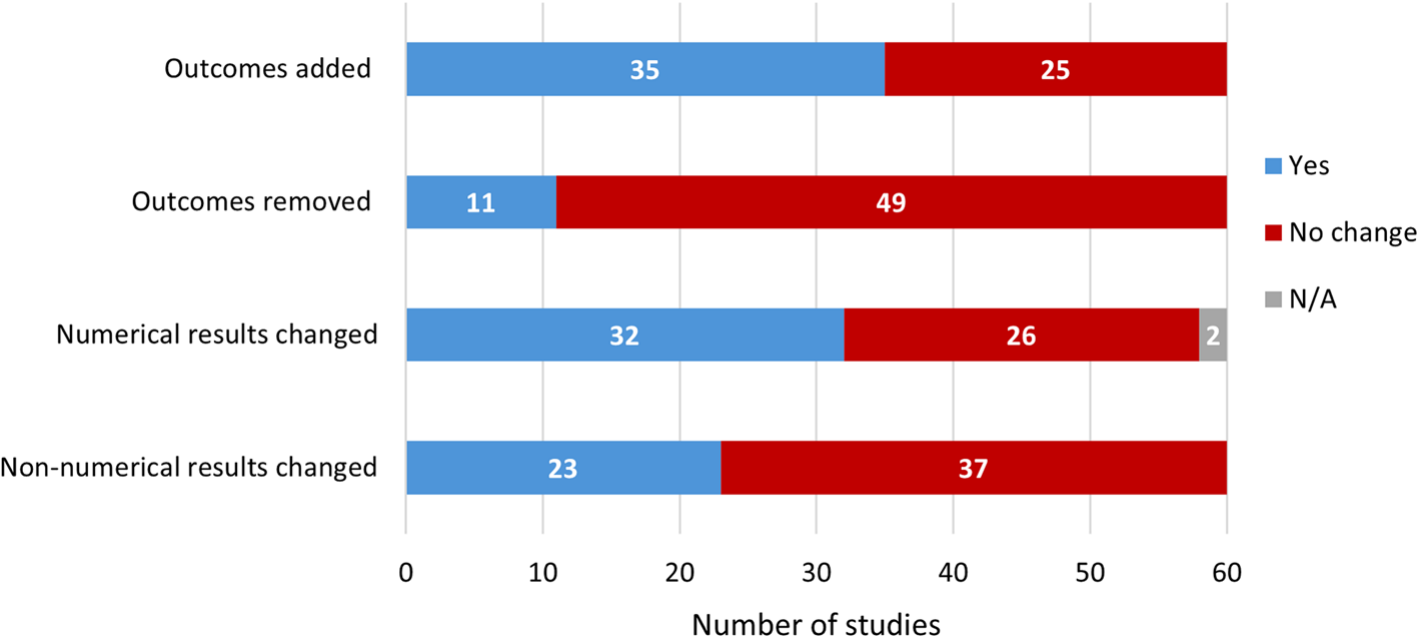
Changes in outcomes and results from the preprint to published version (*N* = 60). For changes in numerical results, studies that did not report any numerical results were labelled as N/A

Outcomes were often added (58%; 35/60) and sometimes removed (18%; 11/60) from the published version (Fig. 2).

Just over half the studies (53%; 32/60) had a change in numerical results from the preprint to published version (Fig. 2). However, among these studies the majority (69%; 22/32) had changes that were minor and did not impact the main conclusions of the study. For example, in one in vitro study the homology between A33 proteins changed from 92.68% in the preprint to 93.5% in the published version [123, 124]. In 82% (18/22) of studies with minor changes in numerical results, there was at least one change in the methods. For a breakdown of the impact of numerical results changes on main conclusions and the associated methods changes, see Additional file 4: Supplementary Table 2.

Slightly more impactful changes in numerical results softened or strengthened the main conclusions in 25% (8/32) of studies. For example, in one predictive modelling study, the preprint reported that 10 to 10,000 additional cases of mpox may be observed if a substantial number of infections are introduced into a specific population, while the published version only reported 10 to 3000 additional cases [125, 126]. In this case, a change in the statistical analysis resulted in a change to the magnitude of the main conclusion, rather than the direction. These changes in magnitude occurred for several predictive or mathematical model studies (75%; 6/8).

Three observational studies (two cross-sectional, one cohort) had changes in numerical results that caused a reversal of the main conclusions. Two of these studies had changes in statistical analysis and other methods, while one had changes across all methods categories. In one cross-sectional preprint, a main conclusion was that participants had “insufficient” knowledge of mpox, but in the published version this changed to “sufficient” knowledge [19, 20]. This change was likely due to the changes in survey methods and statistical analysis. In the published version, a survey question was removed, the scoring system for responses was changed, and one mean knowledge score was reported for all questions combined, rather than two scores for two groups of questions that were presented in the preprint. In the other cross-sectional preprint, a main conclusion was that 69% of respondents intended to probably or definitely reduce their number of sexual partners and 78% intended to probably or definitely have less sex during the mpox epidemic, but in the published version these percentages were reversed to 31% and 22%, respectively [29, 36]. In the cohort preprint, previous syphilis infection was not associated with mpox in univariate analysis so it was not included in the multivariate analysis, however in the published version there was an association in multivariate analysis and syphilis was concluded to be a risk factor for mpox infection [33, 37]. This study also underwent numerical changes to all of the hazard ratios reported in the multivariate analysis, which softened or strengthened main conclusions related to other risk factors.

38% (23/60) of studies had changes in non-numerical results that impacted the main conclusions of the study (Fig. 2). These changes largely consisted of adding to the main conclusions or providing additional evidence for them, rather than altering them. For example, in one cross-sectional study, risk perception was added as an outcome to the published version, which resulted in a new main conclusion that risk perception had a strong positive association with mpox vaccination intentions [25, 26]. Non-numerical changes in results also included adding or updating figures and tables that supported main conclusions.

## Discussion

This paper focuses on the role of preprints in evidence-informed decision-making, particularly when evidence is urgently needed during a public health emergency. Preprint servers for research in disciplines such as mathematics, physics, and biology have been used for years [82, 83], as they circumvent challenges with the peer-review process including high associated costs, lengthy review time, and potential reviewer biases [127, 128]. However, the credibility of preprints remains a concern especially when the results are to be used for public health or medical decision-making [129], such as results from clinical trials on vaccines or therapeutics [130]. Preprints can also be removed from the preprint server [131], which is problematic if their results have been used but the source paper is no longer available; when peer-reviewed articles are retracted, the paper is still available in the journal with a retraction notice for transparency [132].

During the COVID-19 pandemic, there was a sharp increase in the posting and use of preprints in public health and medical research due to the urgent need for timely data. Many researchers posted on a preprint server for the first time as preprint servers were flooded with COVID-19 research, which represented ~ 25% of all COVID-19 literature in October 2020 [133]. Even with accelerated publishing efforts by journals, the data in preprints was available an average of two months earlier than the published version [4, 133]. Findings were similar for mpox; 24% of the primary research in the evidence surveillance database remained a preprint six months into the outbreak [7]. This suggests that during a public health emergency approximately a quarter of the available evidence would be missing without the preprint mechanism. This evidence is invaluable to the public health response, particularly when there are many knowledge gaps or the situation is changing. As demonstrated by this study’s findings and previous studies [4–6], preprints are a resource that is comparable in terms of quality and data to published literature.

Examination of the mpox preprints in this study identified minimal differences in quality between preprints and their published versions. Quality was low across all study designs, regardless of publication status. This may be due to the descriptive nature of many studies produced at the beginning of the outbreak, which are inherently at high risk of bias. Furthermore, the observational studies were rapidly conducted using methods that required less time but increased risk of bias in the results, such as by relying on convenience samples and not controlling for confounders. Given these descriptive and observational study designs, quality remained low even after going through the peer-review process. The comparison of quality between unpublished and published preprints was limited by the small sample sizes for each study design. However, there were no major differences in overall quality. Most of the changes in numerical results between preprint to published did not impact the main conclusions of the study; these changes could be attributed to minor changes in methods, or potential errors in reporting or the dataset. The addition of outcomes in many published studies may have been due to authors performing additional analyses in response to feedback from peer-reviewers. In a few instances, details in the methods and results were removed when preprints were published as different article types with shorter word limits (e.g. letter to the editor). Overall, while the peer-review process did not largely impact the quality of a paper produced during the beginning of a public health emergency, it resulted in additional outcomes and evidence to support main conclusions, and likely improved the accuracy of numerical results. The impact of adding and removing data between preprint and publication deserves more research into why this occurs and its importance.

Previous studies comparing COVID-19 preprints to their published versions have found minimal changes in methods, outcomes, general reporting characteristics, and main conclusions in abstracts [4–6]. One of these studies found that 36% of papers had discrepancies in numerical results, although there was no assessment of how these changes impacted main conclusions [4]. Another study found that the majority of changes to abstracts did not “qualitatively change the conclusions of the paper” [5], which aligns with the results from this study when examining changes in the full text. Thus, these findings suggest that preprints can be used for decision-making during a public health emergency as a good representation of what the published version will be.

### Strengths and limitations

The current research is a comprehensive examination of all preprints released at the onset of a public health emergency compared to their published versions, by examining all study designs and assessing their quality as well as the impact of changes in results on main conclusions.

One limitation is that several study designs did not undergo a quality assessment as there were no appropriate tools (e.g., cluster investigations, in vitro, and predictive models). Furthermore, when reviewers completed quality assessment, they could not be blinded as to whether the paper was a preprint or had been published. A reviewer may have had an unconscious bias towards rating the preprint as lower quality or may have remembered responses for one version when completing the other.

Another limitation is the subjectivity involved in assessing whether the changes in numerical results had an impact on the main conclusions. This issue was mitigated by instructing the reviewer to include relevant explanations and having a second reviewer verify responses.

Finally, these findings only apply to preprints that were produced during the onset of a public health emergency, most of which were low quality even after being published. Findings may be different for higher-quality preprints produced during non-emergency times and their published versions, which was beyond the scope of this study.

## Conclusions

This research examined the quality, and changes in general reporting characteristics, abstracts, methods, outcomes, and results between mpox preprints and their subsequent published versions. Overall quality was comparable between preprints and their published counterparts, with quality generally assessed as low across all study designs based on criteria in validated tools. There were no patterns identified to distinguish between unpublished and published preprints. The majority of changes in numerical results from preprint to published did not impact the main conclusions of the study. Only a few changes impacted the magnitude of the main conclusions, and three changes reversed the studies’ conclusions. The addition of information, and in some cases outcomes, in the published version were considered to be normal products of the peer-review process. Given that these did not impact the overall consistency in main conclusions between preprint and published versions, preprints were considered reliable sources of new scientific evidence for decision-making during public health emergencies.

## Supplementary Information

Additional file 1: Protocol.

Additional file 2: Changes in data. Dataset with results from the data extraction comparing preprints to published versions.

Additional file 3: Quality assessment. Dataset with results from the quality assessment, categorized by study design.

Additional file 4: Supplementary tables. Supplementary Table 1 Summary of changes in general reporting characteristics between preprints and published versions (*N *= 60). Supplementary Table 2 Summary of changes in numerical results from preprint to published versions, and associated methods changes (*N* = 32).

## Data Availability

The datasets generated and analysed during the current study are available in Additional file 2 and 3.

## Abbreviations

NOS: Newcastle-Ottawa Scale
QUADAS-2: Quality Assessment of Diagnostic Accuracy Studies 2
ROB: Risk of bias
