# Supplementary material for: An evaluation of the preprints produced at the beginning of the 2022 mpox public health emergency
Source: Res Integr Peer Rev. 2024 Oct 7;9:11. doi: 10.1186/s41073-024-00152-w (PMC11457328; doi:10.1186/s41073-024-00152-w)
Supplement: Supplementary file 1 — Additional file 1: Protocol. [file 41073_2024_152_MOESM1_ESM.docx]

An evaluation of the preprints produced at the beginning of the 2022 mpox public health emergency: Protocol

Authors: Melanie Sterian ^1^, Anmol Samra ^1,2^, Kusala Pussegoda ^1^, Tricia Corrin ^1^, Mavra Qamar ^1^, Austyn Baumeister ^1^, Izza Israr ^1,2^, Lisa Waddell ^1^

^1^Public Health Risk Sciences Division, National Microbiology Laboratory, Public Health Agency of Canada, Guelph, Canada

^2^Department of Population Medicine, University of Guelph, Guelph, Canada

Important Dates

Evidence published up to June 30, 2023

Protocol version 1 initiated on June 2, 2023

Protocol version 1 finalized on July 31, 2023

Protocol version 2 initiated on August 10, 2023 to reflect changes made after data extraction pilot

Protocol version 2 updated on January 12, 2024 to remove one criteria from the JBI Checklist for Prevalence Studies

Protocol version 2 updated on August 1, 2024 to update the search date for preprint publication status

Table of Contents

[Background 2](#_Toc138400761)

[Objectives 2](#_Toc138400762)

[Research Question(s) 2](#_Toc138400763)

[Planned Study Outputs 3](#_Toc138400764)

[Methods 3](#_Toc138400765)

[Information sources and search strategy 3](#_Toc138400766)

[Eligibility criteria 3](#_Toc138400767)

[Inclusion criteria 3](#_Toc138400768)

[Exclusion criteria 4](#_Toc138400769)

[Data management 4](#_Toc138400770)

[Selection process 4](#_Toc138400771)

[Data characterization 4](#_Toc138400772)

[Quality assessment 5](#_Toc138400773)

[Data synthesis 6](#_Toc138400774)

[Appendix 1: Quality Assessment Form 6](#_Toc138400775)

[Appendix 2: Data Characterization Form 7](#_Toc138400776)

[Appendix 3: Quality Assessment Tools 11](#_Toc138400777)

[3.1 Newcastle-Ottawa Scale adapted for cross-sectional studies 11](#_Toc138400778)

[3.2 Newcastle-Ottawa Scale for case-control studies 12](#_Toc138400779)

[3.3 Newcastle-Ottawa Scale for cohort studies 14](#_Toc138400780)

[3.4 QUADAS-2 16](#_Toc138400781)

[3.5 JBI Critical Appraisal Checklist for prevalence studies 18](#_Toc138400782)

[3.6 JBI Critical Appraisal Checklist for case reports 18](#_Toc138400783)

[3.7 JBI Critical Appraisal Checklist for case series 19](#_Toc138400784)

[References 22](#_Toc138400785)

# Background

Preprints are scientific manuscripts that are shared openly before undergoing the formal peer review process. They are a means of rapidly disseminating new research findings to the scientific community, as well as the general public. They are especially important in the context of public health emergencies, as timely access to preliminary research can enable scientists, policymakers, and healthcare providers to respond better to widespread emergencies (Guterman & Braunstein, 2020).

The mpox (previously known as monkeypox) outbreak began in May 2022 and was declared a Public Health Emergency of International Concern by the World Health Organization on July 23, 2022 (WHO, 2023a). Mpox is a zoonotic disease caused by the monkeypox virus, with a reported count of 87,979 confirmed cases worldwide as of June 12, 2023 (WHO, 2023a). The disease spreads through close or intimate contact and symptoms include fever, respiratory symptoms, and rashes (WHO, 2023b).

As it has been more than a year since the beginning of the mpox outbreak in May 2022, it is now possible to assess the publication status of preprints released during the initial outbreak and compare preprints to their published versions. Previous research has examined discrepancies between COVID-19 preprints and their subsequent journal publications, such as changes in outcomes reported, methods, main conclusions in the abstract, and study characteristics (Bero et al., 2021; Brierley et al., 2022; Spungen et al., 2023). Currently, there is no research investigating preprints that were released at the onset of the 2022-2023 monkeypox outbreak and their published versions.

Objectives

The objective of this study is to evaluate the utility of preprints for decision-making during a public health emergency, by examining the data and quality of preprints released at the onset of the 2022 mpox outbreak. The data examined will include study characteristics, methods, outcomes, and abstracts. Mpox preprints posted from May to August 2022 will be followed through to July 2024 (formerly June 2023) for their publication status. The goal is to understand whether the data and quality of preprints differs from the published versions, and whether the quality of the preprints that were eventually published differs from those that were not.

# Research Question(s)

1. Among the preprints that were posted between May to August 2022 and published by July 2024, do potential changes in quality or data between the preprint and published version impact the main conclusions of the paper?
2. Are there differences in quality between preprints that were published compared to those that were not?

# Planned Study Outputs

1. A paper evaluating preprints and their published versions

# Methods

This study will be conducted by individuals with knowledge synthesis expertise at the Public Health Agency of Canada. AS and MK will serve as project leads, which involves developing the protocol, synthesizing data and writing the manuscript. LW will provide methodological guidance while TC, KP, MQ, AB, and II will provide feedback on the protocol, perform data characterization, and review the manuscript.

Information sources and search strategy

A database of all mpox literature was established in May 2022, as part of a living evidence profile in response to the 2022 mpox outbreak. A comprehensive search strategy was developed and tested through an iterative process by an experienced information specialist in consultation with the review team and peer-reviewed by international colleagues. PubMed, Scopus, EuropePMC, SSRN and arXiv were searched twice weekly between 1 May 2022 to December 31, 2022 to identify preprint and published literature on mpox. The searches utilized keywords (e.g., monkeypox, mpox, simianpox, MPXV, variole du singe, and variole simienne). There were no restrictions on language, however the search was constructed using English and French terms for mpox. Results were limited to the period of 14 April 2022 which was one month prior to the outbreak and 31 December 2022. The search algorithms were adapted to each respective database. Since May 2022, results of all primary and non-primary literature were maintained in RefWorks, DistillerSR (Evidence Partners, 2023) and a searchable excel database, referred to as the mpox database. Each citation in the mpox database was categorized according to literature type (primary or non-primary), study design, and publication status (preprint or published). For this study, eligible mpox preprints were identified by filtering these categories.

## Eligibility criteria

All citations in the mpox database will be assessed for inclusion based on the following eligibility criteria.

### Inclusion criteria

STUDY DESIGN: Primary studies posted as preprints will be included.

ARTICLE TYPE: Only the first version of a preprint will be included (when it was first available online), as the first version is most likely to have been viewed by users and later versions may have been changed in response to feedback from peers (Bero et al., 2021).

PUBLICATION RANGE: Preprints posted between May 1, 2022 and August 22, 2022, because this indicates the last time before which the global mpox case count consistently starts to decline, according to data from the World Health Organization (WHO, 2023a). Preprints will be followed through to June 2023 for publication status, which is 9 to 12 months after the preprint was first released.

POPULATION: Primary studies evaluating any population.

INTERVENTION: Primary studies evaluating any intervention/exposure or no intervention.

COMPARATORS: Primary studies with or without a comparator group.

OUTCOMES: Primary studies reporting any outcome.

LANGUAGE: Primary studies in English and French.

### Exclusion criteria

Non-primary studies (e.g. reviews) and methods studies will be excluded because these studies do not provide primary results that could be compared between a preprint and published version.

Data management

All eligible preprint citations and their published versions will be exported from RefWorks and uploaded to DistillerSR. Distiller SR will be used to manage quality assessment and data characterization of citations.

Selection process

In order to select preprints that were released at the onset of the mpox outbreak, the mpox database will be filtered by preprint posting date of May 1, 2022 to August 22, 2022. The mpox database will also be filtered for primary literature, excluding methods studies. To identify published versions of preprints, the duplicate log in the mpox database will be used. Additionally, in June 2023 and updated in July 2024, each article labeled as a preprint in the mpox database will be manually screened to assess if it has been published. This will be done by checking for indications of publication directly on the preprint article page or by manually searching on Google or Google Scholar for the published version of the article. Finally, the identified preprints and published versions will be pre-screened to ensure that the language is English or French before citations are uploaded to DistillerSR for data characterization and quality assessment.

# Data characterization

A data characterization form to extract relevant information from the studies is provided in Appendix 2. The form will be pilot tested by all reviewers on a random sample of five to ten articles and adjusted as needed. Data characterization will be performed in DistillerSR using an accelerated process of reviewing, in which a senior reviewer verifies the extraction form completed by a junior reviewer.

Some data of interest, including country of conduct, study design, and preprint posting date, has already been extracted for the living evidence profile and is therefore not included in the data characterization form. Additionally, publishing characteristics of interest, including preprint server and journal name, are already captured in the reference information in Distiller so these are not included in the data extraction form. Reviewers will complete one data characterization form for each preprint-published pair. The reviewer will look at the preprint and published version at the same time when completing the form. Disagreements will be resolved by consensus or by consulting with a third reviewer.

The form includes questions regarding the following: (1) general study characteristics, such as funding source and conflict of interest statements, (2) changes in methods, such as in the statistical analysis or sample size, (3) changes in results (e.g. number of outcomes reported, statistical significance of outcome), and (4) changes in the main conclusion of the abstract.

General Study Characteristics

- Publication status of preprint (published or unpublished)
- Changes in the author list from preprint to published version
- Changes in funding statement from preprint to published version
- Changes in conflict of interest statement from preprint to published version
- Publication date of published article

Abstract

- Changes in the results the authors chose to highlight in abstract from preprint to published version

Methods

- Total sample size of the population
- Study period
- Statistical analysis
- Any other methods changes

Outcomes and Results

- Outcomes removed
- Outcomes added
- Any numerical results that changed
- Impact of changed numerical results on main conclusions of study
- Any other changes in results (not numerical) that impact main conclusions of study

# Quality assessment

While data will be extracted for all study designs, quality assessment will only be conducted for the following study designs: case-control, cohort, cross-sectional, diagnostic test accuracy studies, surveillance data analyses, case reports and case series. Quality assessment will be performed in duplicate on Distiller. For preprint-published pairs, two quality assessment forms will be completed: one for the preprint and one for the published version.

The quality assessment tools that will be used include:

- Newcastle-Ottawa Scale for case-control studies (G. Wells et al.)
- Newcastle-Ottawa Scale for cohort studies (G. Wells et al.)
- Adapted version of the Newcastle-Ottawa Scale for cross-sectional studies (Herzog et al., 2013; Ribeiro et al., 2020)
- Quality Assessment of Diagnostic Accuracy Studies 2 (QUADAS-2) (Whiting, 2011)
- JBI Critical Appraisal Checklist for prevalence studies (Munn et al., 2015)
  - This Checklist will be used to assess quality of surveillance data analyses. One criteria assessing adequate sample size was removed from the Checklist, as it was not applicable for surveillance data analyses.
- JBI Critical Appraisal Checklist for case reports (Moola S, 2020)
- JBI Critical Appraisal Checklist for case series (Munn et al., 2020)

A validated quality assessment tool was not available for the remaining study designs: cluster investigations, in vitro, in silico, phylogenetic analyses, exposure investigations, and mathematical and predictive epidemiological models. Therefore, quality assessment will not be performed for articles with these study designs.

# Data synthesis

Data including the study characteristics, publishing characteristics (e.g. preprint server, journal), changes in methods, changes in results, and quality assessments will be synthesized narratively and summarized using descriptive statistics (e.g., frequency, percentage, and proportions) as well as groupings of categories (e.g., study designs). No formal statistical analysis will be conducted. When necessary, a graphical display of the evidence will be provided in figures and tables.

Quality will be reported across subgroups of study designs, for example comparing changes in quality between preprints and published versions for all cohort studies.

# Appendix 1: Quality Assessment Form

| **#** | **Question** | **Options** | **Comments** |
| --- | --- | --- | --- |
| **Quality Assessment Part 1** | | | |
| 1 | What is the study design of this paper? | - Cross-sectional - Case control - Cohort - Case report - Case series - Mathematical model - Predictive model - In silico - In vitro - Diagnostic test accuracy - Phylogenetic analysis report - Surveillance data analysis - Cluster investigation - Exposure investigation - Bioinformatic analysis | A label will be attached to each form indicating the article’s study design (based on prior extraction in the mpox database). If you believe the study design of this paper does not match the study design label provided, please notify the lead and do NOT proceed to the quality assessment until study design has been verified.  If cross-sectional, case-control, cohort, surveillance data analysis, case report, case series, or diagnostic test accuracy is selected, then Part 2 appears with the relevant quality assessment tool depending on the study design.  For any other response, no further questions will appear and the reviewer will be instructed to submit the form. |
| **Quality Assessment Part 2** | | | |
| 2 | Questions for the relevant quality assessment tool will appear based on answer in Part 1. See Appendix 3 for the specific questions used in each tool. | If cross-sectional, the NOS adapted cross-sectional version appears.  If case control, the NOS for case control studies appears.  If cohort, the NOS for cohort studies appears.  If surveillance data analysis, the JBI Critical Appraisal Checklist for prevalence studies appears.  If case report or case series, the applicable JBI Critical Appraisal Checklist appears.  For diagnostic test accuracy, QUADAS-2 appears. |  |

# Appendix 2: Data Characterization Form

| **#** | **Question** | **Options** | **Comments** |
| --- | --- | --- | --- |
| **General Study Characteristics** | | | |
| 1 | Was the preprint published? | - YES - NO | A label will be attached to each form indicating the preprint’s publication status.  If YES is selected, then the following questions will appear. If NO is selected, the reviewer will be instructed to submit the form. |
| 2 | Has the author list changed from the preprint to the published version? Check all that apply. | - YES, authors added [TEXT] - YES, authors removed [TEXT] - NO | In the textbox, specify the number of authors added or removed. |
| 3 | Has the funding statement changed from the preprint to published version? | - YES, higher number of funding sources provided in journal publication [TEXT] - YES, fewer number of funding sources provided in journal publication [TEXT] - YES, any other changes [TEXT] - NO - N/A (only reported in one version, or not reported in either) | In the textbox, specify the funding sources that were added or removed and include any relevant explanations for changes. |
| 4 | Has the conflict of interest statement changed from the preprint to the published version? | - YES [TEXT] - NO - N/A (only reported in one version, or not reported in either) | In the textbox, explain how the COI has changed (e.g. more conflicts reported) and include any relevant explanations for change (e.g. author list change). |
| 5 | What is the date that the published version was first released? | [TEXT] | Provide the full date in the following format: DD month YYYY (e.g., 12 June 2023).  Capture the epub date (when the article first went live online). |
| **Change in Abstract** | | | |
| 6 | In the abstract, were there any changes in the results the authors chose to highlight? | - NO change, authors highlighted the same results in the abstract. - YES, authors chose to highlight different results in the abstract. [TEXT] - N/A (abstract only provided in one version, or not provided in either) | If YES, please describe how the results highlighted by the authors changed (e.g. a result was omitted in the abstract of published). |
| **Methods** | | | |
| 7 | Has the total sample size of the sample population changed in the published version? | - YES: [TEXT] - NO - N/A | If YES, write the sample size for preprint and published and include any relevant explanations why it changed. |
| 8 | Has the study period changed in the published version? | - YES: [TEXT] - NO - N/A | If a study period is not reported, select N/A. |
| 9 | Has the statistical analysis changed in the published version? | - YES: [TEXT] - NO - N/A | If YES, broadly explain how the statistical analysis has changed in the published version (e.g. statistical test, model, confounders). If sensitivity and/or subgroup analyses has changed, explain in textbox as well. |
| 10 | Did any other methods change in the published version? | - YES [TEXT] - NO | For changes in methods that are not already captured by the previous questions, briefly describe these changes in the textbox.  If some methods have been moved from supplementary to main text or vice versa, this is not considered a change in methods. |
| **Outcomes and Results** | | | |
| 11 | Were any outcomes removed in the published version? If yes, specify which. | - YES: [TEXT] - NO | Please look at both the main text and the supplementary material for the outcomes.  If YES, specify which outcome was omitted and the reason for omitting if known. |
| 12 | Were any outcomes added in the published version? If yes, specify which. | - YES: [TEXT] - NO | Please look at both the main text and the supplementary material for the outcomes.  If YES, specify which outcome was added and the reason for adding if known. |
| 13 | Have any numerical results for primary outcomes changed from preprint to published? Only look at numerical results reported in both preprint and published versions. | - YES - NO - N/A (no numerical results were reported) | If YES, question 14 appears. |
| 14 | Does the change in numerical results impact the main conclusion(s) of the study? Check all that apply. | - NO impact – minor change in numerical results does not impact main conclusion(s). [TEXT] - YES, major impact – change in numerical results leads to a softening or strengthening of main conclusion(s). [TEXT] - YES, massive impact – change in numerical results leads to a reversal of main conclusion(s). [TEXT] | In textbox, specify for which outcome the results changed and include relevant explanations on why changes in numerical results did or did not impact main conclusions.  e.g. there was no impact on main conclusions because only the 95%CI slightly changed.  e.g. there was a massive impact because the p-value and 95%CI changed so that a non-significant association has now become significant. |
| 15 | Are there any other changes in results (not numerical) that impact the main conclusion(s) of the study? | - YES: [TEXT] - NO | Briefly describe any other changes not already captured that impact main conclusions – for example: results for additional outcomes that add to main conclusions, additional figures in results that support the main conclusions, or changes in qualitative results that strengthen/soften main conclusions. |

# Appendix 3: Quality Assessment Tools

## 3.1 Newcastle-Ottawa Scale adapted for cross-sectional studies

| **Question** | **Options** | **Comments** |
| --- | --- | --- |
| **Selection (maximum 3 points)** | | |
| Representativeness of the sample | - Truly representative of the average in the target population (**1 POINT) [TEXT] - Somewhat representative of the average in the target population [TEXT] - Selected group of users (e.g. nurses, volunteers) [TEXT] - No description of the sampling strategy [TEXT] | Note to the rater: For each rating, paste supporting text into the comment box unless the rating is not reported. |
| Sample size | - Sample size justified and satisfactory (**1 POINT) [TEXT] - Sample size not justified or reported [TEXT] |  |
| Non-included subjects | - Comparability between included and non-included subjects was established, and the inclusion rate was satisfactory (**1 POINT) [TEXT] - Comparability between included and non-included subjects not established and not satisfactory [TEXT] |  |
| **Comparability (maximum 2 points)** | | |
| The subjects in different outcome groups are comparable, based on the study design or analysis. Confounding factors are controlled. | - Study controls for age and sex (**1 POINT) [TEXT] - Study controls for other factors (e.g. race, ethnicity, SES) (**1 POINT) [TEXT] - Not reported or no factors controlled for [TEXT] |  |
| **Outcome (maximum 3 points)** | | |
| Assessment of the outcome | - Independent blind assessment (**1 POINT) [TEXT] - Medical records [TEXT] - Self-report (**1 POINT) [TEXT] - No description [TEXT] |  |
| Statistical test | - Statistical test used was clearly described and appropriate, and the measurement of the association was presented with confidence interval and p-value (**1 POINT) [TEXT] - Statistical test was not appropriate, not described or incomplete [TEXT] |  |
| **Total Quality Score** | | |
| Total Quality Score | Automatically calculated for a total score ranging from 0 to 9 points. |  |

## 3.2 Newcastle-Ottawa Scale for case-control studies

| **Question** | **Options** | **Comments** |
| --- | --- | --- |
| **Selection (maximum 4 points)** | | |
| Is the case definition adequate? | o Yes, with independent  validation (e.g. >1  person/record/time/process to  extract information) (**1  POINT) [TEXT]  o Yes (e.g. medical records such  as ICD codes in database or  based on self reports with no  reference to a primary record)  [TEXT]  o No description of how a case is defined [TEXT] | Note to the rater: For each rating, paste supporting text into the comment box unless the rating is not reported. |
| Are the cases representative? | o Consecutive or obviously  representative series of cases  (**1 POINT) [TEXT]  o Potential for selection biases  [TEXT]  o Not stated [TEXT] |  |
| Are the selection of controls from the same community? | o Community controls (i.e. same  community as cases and would  be cases if they had the  outcome) (**1 POINT) [TEXT]  o Hospital controls (i.e. within  same community as cases but  derived from a hospitalized  population) [TEXT]  o Controls were selected from  another population or no  description of how controls  were selected [TEXT] |  |
| Definition of controls | o Controls have no history of the outcome/disease/endpoint. If cases are first occurrence of the outcome of interest, then it  must explicitly state that  controls have no history of this  outcome. If cases have new  (not necessarily first)  occurrence of outcome, then  controls with previous  occurrences of outcome of  interest should not be excluded.  (**1 POINT) [TEXT]  o No mention of history of  outcome/disease/endpoint for  the controls [TEXT] |  |
| **Comparability (maximum 2 points)** | | |
| Comparability of cases and controls on the basis of the design or analysis. | o Study controls for age and sex  (**1 POINT) [TEXT]  o Study controls for other factors (e.g. race, ethnicity, SES) (**1 POINT) [TEXT]  o Not reported or no controls  [TEXT] | Note to reviewer: Either cases and controls must be matched in the design and/or confounders must be adjusted for in the analysis. Statements of no differences between groups or that differences were not statistically significant are not sufficient for establishing comparability. |
| **Exposure (maximum 3 points)** | | |
| Ascertainment of exposure | o Secure record (e.g. surgical  records) (**1 POINT) [TEXT]  o Structured interview where  blind to case/control status  (**1 POINT) [TEXT]  o Interview not blinded to  case/control status [TEXT]  o Written self report or medical  record only [TEXT]  o No description [TEXT] |  |
| Same method of ascertainment for cases and controls | o Yes (**1 POINT) [TEXT]  o No [TEXT] |  |
| Non-response rate | o Same rate for both groups (**1 POINT) [TEXT]  o Non-respondents described  [TEXT]  o Non-response rate different  and no designation [TEXT] |  |
| **Total Quality Score** | | |
| Total Quality Score | Automatically calculated for a total score ranging from 0 to 9 points. |  |

## 3.3 Newcastle-Ottawa Scale for cohort studies

| **Question** | **Options** | **Comments** |
| --- | --- | --- |
| **Selection (maximum 4 points)** | | |
| Representativeness of the exposed cohort | o Truly representative of the  average in the community (**1  POINT) [TEXT]  o Somewhat representative of  the average in the community  [TEXT]  o Selected group of users (e.g.  nurses, volunteers) [TEXT]  o No description of the derivation  of the cohort [TEXT] | Note to the rater: For each rating, paste supporting text into the comment box unless the rating is not reported. |
| Selection of the non-exposed  cohort | o Drawn from the same  community as the exposed  cohort (**1 POINT) [TEXT]  o Drawn from a different source  [TEXT]  o No description of the derivation  of the non-exposed cohort  [TEXT] |  |
| Ascertainment of exposure | o Secure record (e.g. hospital  records) (**1 POINT) [TEXT]  o Structured interview [TEXT]  o Written self-report [TEXT]  o No description [TEXT] |  |
| Demonstration that outcome of  interest was not present at start of study | o Yes (**1 POINT) [TEXT]  o No [TEXT] |  |
| **Comparability (maximum 2 points)** | | |
| Subjects in different outcome groups are comparable, based on study design or analysis. Confounding factors are controlled. | o Study controls for age and sex (**1 POINT) [TEXT]  o Study controls for other factors (e.g. race, ethnicity, SES) (**1 POINT) [TEXT]  o Not reported or no control [TEXT] |  |
| **Outcome (maximum 3 points)** | | |
| Assessment of outcome | o Independent blind assessment  (**1 POINT) [TEXT]  o Medical records [TEXT]  o Self-report [TEXT]  o No description [TEXT] |  |
| Was follow-up long enough for outcomes to occur? | o Yes (**1 POINT) [TEXT]  o No [TEXT] |  |
| Adequacy of follow-up of cohorts | o Complete follow-up, all subjects accounted for (**1 POINT) [TEXT]  o Subjects lost to follow-up (0-  25%) unlikely to introduce bias.  [TEXT]  o Follow-up rate less than 75%  and no description of those lost.  [TEXT]  o No statement regarding follow-up of cohorts. [TEXT] | Include the follow-up rate (percentage) in the text box. |
| **Total Quality Score** | | |
| Total Quality Score | Automatically calculated for a total score ranging from 0 to 9 points. |  |

## 3.4 QUADAS-2

| **DOMAIN** | **PATIENT SELECTION** | **INDEX TEST** | **REFERENCE STANDARD** | **FLOW AND TIMING** |
| --- | --- | --- | --- | --- |
| **Description** | Describe methods of patient selection: [TEXT] | Describe the index test and how it was conducted and interpreted: [TEXT] | Describe the reference standard and how it was conducted and interpreted: [TEXT] | Describe any patients who did not receive the index test(s) and/or reference standard or who were excluded from the 2x2 table (refer to flow diagram): [TEXT] |
|  | Describe included patients (prior testing, presentation, use of index test and setting): [TEXT] |  |  | Describe the time interval and any interventions between index test(s) and reference standard: [TEXT] |
| **Signalling questions** | Was a consecutive or random sample of patients enrolled?   - Yes - No - Unclear | Were the index test results interpreted without knowledge of the results of the reference standard?   - Yes - No - Unclear | Is the reference standard likely to correctly classify the target condition?   - Yes - No - Unclear | Was there an appropriate interval between index test(s) and reference standard?   - Yes - No - Unclear |
|  | Was a case-control design avoided?   - Yes - No - Unclear | If a threshold was used, was it pre-specified?   - Yes - No - Unclear | Were the reference standard results interpreted without knowledge of the results of the index test?   - Yes - No - Unclear | Did all patients receive a reference standard?   - Yes - No - Unclear |
|  | Did the study avoid inappropriate exclusions?   - Yes - No - Unclear |  |  | Did all patients receive the same reference standard?   - Yes - No - Unclear |
|  |  |  |  | Were all patients included in the analysis?   - Yes - No - Unclear |
| **Risk of bias** | Could the selection of patients have introduced bias?   - High - Low - Unclear | Could the conduct or interpretation of the index test have introduced bias?   - High - Low - Unclear | Could the reference standard, its conduct, or interpretation have introduced bias?   - High - Low - Unclear | Could the patient flow have introduced bias?   - High - Low - Unclear |
| **Concerns regarding applicability** | Are there concerns that the included patients do not match the review question?   - High - Low - Unclear | Are there concerns that the index test, its conduct, or interpretation differ from the review question?   - High - Low - Unclear | Are there concerns that the target condition as defined by the reference standard does not match the review question?   - High - Low - Unclear |  |

## 3.5 JBI Critical Appraisal Checklist for prevalence studies

| **Question** | **Options** | **Comments** |
| --- | --- | --- |
| Was the sample frame appropriate to address the target population? | - Yes - No - Unclear - Not applicable | The sample frame is the source from which the study participants were drawn. |
| Were study participants sampled in an appropriate way? | - Yes - No - Unclear - Not applicable | In other words, were the study participants identified from the sample frame in an appropriate way? |
| Were the study subjects and the setting described in detail? | - Yes - No - Unclear - Not applicable |  |
| Was the data analysis conducted with sufficient coverage of the identified sample? | - Yes - No - Unclear - Not applicable |  |
| Were valid methods used for the identification of the  condition? | - Yes - No - Unclear - Not applicable |  |
| Was the condition measured in a standard, reliable way  for all participants? | - Yes - No - Unclear - Not applicable |  |
| Was there appropriate statistical analysis? | - Yes - No - Unclear - Not applicable |  |
| Was the response rate adequate, and if not, was the low response rate managed appropriately? | - Yes - No - Unclear - Not applicable | For studies using surveillance data on mpox cases to estimate incubation period, “response rate” refers to the proportion of cases that had complete data collected and were used in analysis. |

## 3.6 JBI Critical Appraisal Checklist for case reports

| **Question** | **Options** | **Comments** |
| --- | --- | --- |
| Were patient’s demographic characteristics clearly described? | - Yes - No - Unclear - Not applicable | Does the case report clearly describe patient's age, sex, race, medical history, diagnosis, prognosis, previous treatments, past and current diagnostic test results, and medications? |
| Was the patient’s history clearly described and presented as a timeline? | - Yes - No - Unclear - Not applicable |  |
| Was the current clinical condition of the patient on presentation clearly described? | - Yes - No - Unclear - Not applicable | The current clinical condition of the patient should be described in detail including the uniqueness of the condition/disease, symptoms, frequency and severity. |
| Were diagnostic tests or assessment methods and the results clearly described? | - Yes - No - Unclear - Not applicable |  |
| Was the intervention(s) or treatment procedure(s) clearly described? | - Yes - No - Unclear - Not applicable |  |
| Was the post-intervention clinical condition clearly described? | - Yes - No - Unclear - Not applicable |  |
| Were adverse events (harms) or unanticipated events identified and described? | - Yes - No - Unclear - Not applicable |  |
| Does the case report provide takeaway lessons? | - Yes - No - Unclear - Not applicable | Case reports should summarize key lessons learned from a case in terms of the background of the condition/disease and clinical practice guidance for clinicians when presented with similar cases. |
| Comments | [TEXT] |  |

## 3.7 JBI Critical Appraisal Checklist for case series

| **Question** | **Options** | **Comments** |
| --- | --- | --- |
| Were there clear criteria for inclusion in the case series? | - Yes - No - Unclear - Not applicable |  |
| Was the condition measured in a standard, reliable way for all participants included in the case series? | - Yes - No - Unclear - Not applicable | The study should clearly describe the method of measurement of the condition. This should be done in a standard (i.e. same way for all patients) and reliable (i.e. repeatable and reproducible results) way. |
| Were valid methods used for identification of the condition for all participants included in the case series? | - Yes - No - Unclear - Not applicable | If the outcomes were assessed based on existing definitions or diagnostic criteria, then the answer to this question is likely to be yes. If the outcomes were assessed using observer reported, or self-reported scales, the risk of over- or under-reporting is increased, and objectivity is compromised. |
| Did the case series have consecutive inclusion of participants? | - Yes - No - Unclear - Not applicable | For example, a case series that states ‘we included all patients (24) with osteosarcoma who presented to our clinic between March 2005 and June 2006’ is more reliable than a study that simply states ‘we report a case series of 24 people with osteosarcoma.’ |
| Did the case series have complete inclusion of participants? | - Yes - No - Unclear - Not applicable |  |
| Was there clear reporting of the demographics of the participants in the study? | - Yes - No - Unclear - Not applicable | The case series should clearly describe relevant participant’s demographics such as the following information where relevant: participant’s age, sex, education, geographic region, ethnicity, time period, education. |
| Was there clear reporting of clinical information of the participants? | - Yes - No - Unclear - Not applicable | There should be clear reporting of clinical information of the participants such as the following information where relevant: disease status, comorbidities, stage of disease, previous interventions/treatment, results of diagnostic tests, etc. |
| Were the outcomes or follow up results of cases clearly reported? | - Yes - No - Unclear - Not applicable |  |
| Was there clear reporting of the presenting site(s)/clinic(s) demographic information? | - Yes - No - Unclear - Not applicable |  |
| Was statistical analysis appropriate? | - Yes - No - Unclear - Not applicable |  |
| Comments | [TEXT] |  |

#

# References

Bero, L., Lawrence, R., Leslie, L., Chiu, K., McDonald, S., Page, M. J., Grundy, Q., Parker, L., Boughton, S., Kirkham, J. J., & Featherstone, R. (2021). Cross-sectional study of preprints and final journal publications from COVID-19 studies: discrepancies in results reporting and spin in interpretation. *BMJ Open*, *11*(7), e051821. <https://doi.org/10.1136/bmjopen-2021-051821>

Brierley, L., Nanni, F., Polka, J. K., Dey, G., Pálfy, M., Fraser, N., & Coates, J. A. (2022). Tracking changes between preprint posting and journal publication during a pandemic. *PLoS biology*, *20*(2), e3001285.

G. Wells, B. S., D. O’Connell,, J. Robertson, J. P., V., & Welch, M. L., P. Tugwell. *The Newcastle-Ottawa Scale (NOS) for assessing the quality of nonrandomised studies in meta-analyses*. <https://www.ohri.ca/programs/clinical_epidemiology/nosgen.pdf>

Guterman, E. L., & Braunstein, L. Z. (2020). Preprints During the COVID-19 Pandemic: Public Health Emergencies and Medical Literature. *Journal of hospital medicine*, *15*(10), 634-636. <https://doi.org/10.12788/jhm.3491>

Herzog, R., Álvarez-Pasquin, M. J., Díaz, C., Del Barrio, J. L., Estrada, J. M., & Gil, Á. (2013). Are healthcare workers’ intentions to vaccinate related to their knowledge, beliefs and attitudes? a systematic review. *BMC Public Health*, *13*(1), 154. <https://doi.org/10.1186/1471-2458-13-154>

Moola S, M. Z., Tufanaru C, Aromataris E, Sears K, Sfetcu R, Currie M, Lisy K, Qureshi R, Mattis P, Mu P. (2020). *JBI Critical Appraisal Checklist for Case Reports* (M. Z. Aromataris E, Ed.). JBI. <https://jbi.global/sites/default/files/2019-05/JBI_Critical_Appraisal-Checklist_for_Case_Reports2017_0.pdf>

Munn, Z., Barker, T. H., Moola, S., Tufanaru, C., Stern, C., McArthur, A., Stephenson, M., & Aromataris, E. (2020). Methodological quality of case series studies: an introduction to the JBI critical appraisal tool. *JBI Evidence Synthesis*, *18*(10), 2127-2133. <https://doi.org/10.11124/jbisrir-d-19-00099>

Munn, Z., Moola, S., Lisy, K., Riitano, D., & Tufanaru, C. (2015). Methodological guidance for systematic reviews of observational epidemiological studies reporting prevalence and cumulative incidence data. *JBI Evidence Implementation*, *13*(3), 147-153. <https://doi.org/10.1097/xeb.0000000000000054>

Ribeiro, C. M., Beserra, B. T. S., Silva, N. G., Lima, C. L., Rocha, P. R. S., Coelho, M. S., Neves, F. d. A. R., & Amato, A. A. (2020). Exposure to endocrine-disrupting chemicals and anthropometric measures of obesity: a systematic review and meta-analysis. *BMJ Open*, *10*(6), e033509. <https://doi.org/10.1136/bmjopen-2019-033509>

Spungen, H., Burton, J., Schenkel, S., & Schriger, D. L. (2023). Completeness and Spin of medRxiv Preprint and Associated Published Abstracts of COVID-19 Randomized Clinical Trials. *JAMA*, *329*(15), 1310-1312. <https://doi.org/10.1001/jama.2023.1784>

Whiting, P. F., Rutjes, A.W.S., Westwood, M.E., et al. . (2011). QUADAS-2: A Revised Tool for the Quality Assessment of Diagnostic Accuracy Studies. *Annals of Internal Medicine*. <https://doi.org/https://doi.org/10.7326/0003-4819-155-8-201110180-00009>

WHO. (2023a). *2022-23 Mpox Outbreak: Global Trends*. World Health Organization. Retrieved June 28 from <https://worldhealthorg.shinyapps.io/mpx_global/>

WHO. (2023b). *Mpox (monkeypox)*. World Health Organization. Retrieved June 28 from <https://www.who.int/news-room/fact-sheets/detail/monkeypox>
