# Supplementary material for: An evaluation of the preprints produced at the beginning of the 2022 mpox public health emergency
Source: Res Integr Peer Rev. 2024 Oct 7;9:11. doi: 10.1186/s41073-024-00152-w (PMC11457328; doi:10.1186/s41073-024-00152-w)
Supplement: Supplementary file 4 — Additional file 4: Supplementary tables. Supplementary Table 1 Summary of changes in general reporting characteristics between preprints and published versions (N = 60). Supplementary Table 2 Summary of changes in numerical results from preprint to published versions, and associated methods changes (N = 32). [file 41073_2024_152_MOESM4_ESM.docx]

**Supplementary Table 1.** Summary of changes in general reporting characteristics between preprints and published versions (N=60)

| **Reporting characteristic** | **Studies with change** | **Types of changes** |
| --- | --- | --- |
| Authorship | 23% (14/60) | - Added authors: n=13 (1-26) - Removed authors: n=1 (27, 28) - Removed and added authors: n=1 (1, 2) |
| Funding statement | 28% (17/60) | - Added funding sources: n=6 (13, 14, 29-38) - Removed funding sources: n=2 (3, 4, 17, 18) - Other changes: n=9 (7, 8, 39-54) - e.g. added details on funding source: “institutional funding” in preprint changed to “Medical Biological Defense Research Program of the Bundeswehr Medical Service” in published version |
| Conflict of interest statement | 13% (8/60) | - Added conflicts: n=7 (11, 12, 19-22, 29, 30, 43, 44, 55-58) - Removed conflicts: n=1 (3, 4) |

**Supplementary Table 2.** Summary of changes in numerical results from preprint to published versions, and associated methods changes (N=32)

| **Impact of changes in numerical results on main conclusions** | **Number of associated methods changes in the study** | **Details of methods changes** |
| --- | --- | --- |
| Minor (n=22) | None (n=4) |  |
|  | One (n=4) | - One study had change in sample size (15, 16) - One study had change in study period (although this may be a typo) (59, 60) - One study changed molar ratio to volume ratio (61, 62) - One study had a change in one model assumption (63, 64) |
|  | Multiple (n=14) | - Six studies had a change in statistical analysis (among other changes) (5, 6, 17, 18, 45, 46, 65-70) - Four studies had changes to the dataset used in analysis (among other changes) (9, 10, 13, 14, 17, 18, 27, 28) - Three studies had a change in study period (among other changes) (27, 28, 69-72) |
| Major (n=8) | None (n=1) |  |
|  | One (n=3) | - Two studies had a change in statistical analysis (53, 54, 73, 74) - One mathematical modelling study omitted details about the methodology used to estimate population size (55, 56) |
|  | Multiple (n=4) | - Three studies had changes across all categories (sample size, study design, statistical analysis, and “other methods”) (49, 50, 75, 76) - One predictive modelling study had a change in study period and statistical analysis (25, 26) |
| Massive (n=3) | Multiple (n=3) | - Two studies had changes in statistical analysis and “other methods” (1, 2, 77, 78) - One study had changes across all categories (23, 24) |

## **References**

1. Ahmed SK, Abdulqadir SO, Omar RM, Abdullah AJ, Rahman HA, Hussein SH, et al. Knowledge, Attitude and Worry in the Kurdistan Region of Iraq during the Mpox (Monkeypox) Outbreak in 2022: An Online Cross-Sectional Study. Vaccines. 2023;11(3):610.

2. Ahmed SK, Abdulqadirb SO, Omar RM, Essa RA, Hussein SH, Khdir AA, Abdulla AQ. Study of knowledge, attitude and anxiety in Kurdistan-region of Iraqi population during the monkeypox outbreak in 2022. Research Square; 2022.

3. Allan-Blitz L, Carragher K, Sukhija-Cohen A, Li H, Klausner J. Laboratory Validation and Clinical Implementation of Human Monkeypox Saliva-Based Tests. medRxiv; 2022.

4. Allan-Blitz L-T, Carragher K, Sukhija-Cohen A, Ritchie P, Scott H, Li H, Klausner JD. Laboratory validation and clinical performance of a saliva-based test for monkeypox virus. Journal of Medical Virology. 2023;95(1):e28191.

5. Alshahrani N, Algethami M, Alarifi A, Alzahrani F, Sheerah HA, Abdelaal A, et al. Knowledge and attitude regarding monkeypox virus among physicians in Saudi Arabia, a cross-sectional study. Research Square; 2022.

6. Alshahrani NZ, Algethami MR, Alarifi AM, Alzahrani F, Alshehri EA, Alshehri AM, et al. Knowledge and Attitude Regarding Monkeypox Virus among Physicians in Saudi Arabia: A Cross-Sectional Study. Vaccines. 2022;10(12):2099.

7. De Baetselier I, Van Dijck C, Kenyon C, Coppens J, Michiels J, de Block T, et al. Retrospective detection of asymptomatic monkeypox virus infections among male sexual health clinic attendees in Belgium. Nature Medicine. 2022;28(11):2288-92.

8. De Baetselier I, Van Dijck C, Kenyon C, Coppens J, Van den Bossche D, Smet H, et al. Asymptomatic Monkeypox Virus Infections Among Male Sexual Health Clinic Attendees in Belgium. SSRN - Lancet prepublication. 2022.

9. Gigante CM, Korber B, Seabolt MH, Wilkins K, Davidson W, Rao AK, et al. Multiple lineages of Monkeypox virus detected in the United States, 2021- 2022. bioRxiv. 2022:2022.06.10.495526.

10. Gigante CM, Korber B, Seabolt MH, Wilkins K, Davidson W, Rao AK, et al. Multiple lineages of monkeypox virus detected in the United States, 2021–2022. Science. 2022;378(6619):560-5.

11. Girometti N, Byrne R, Bracchi M, Heskin J, McOwan A, Tittle V, et al. Epidemiological Characteristics and Clinical Features of Confirmed Human Monkeypox Virus Cases in Individuals Attending a Sexual Health Centre in London, United Kingdom. SSRN - Lancet prepublication. 2022.

12. Girometti N, Byrne R, Bracchi M, Heskin J, McOwan A, Tittle V, et al. Demographic and clinical characteristics of confirmed human monkeypox virus cases in individuals attending a sexual health centre in London, UK: an observational analysis. The Lancet Infectious Diseases. 2022;22(9):1321-8.

13. Kumar R, Nagar S, Haider S, Sood U, Ponnusamy K, Dhingra GG, et al. Monkeypox virus: phylogenomics, host–pathogen interactome and mutational cascade. Microbial Genomics. 2023;9(4).

14. Kumar R, Nagar S, Haider S, Sood U, Ponnusamy K, Dhingra GG, et al. Monkey Pox Virus (MPXV): Phylogenomics, Host-Pathogen Interactome, and Mutational Cascade. bioRxiv; 2022.

15. Nörz D, Tang HT, Emmerich P, Giersch K, Fischer N, Addo MM, et al. Rapid adaptation of established high-throughput molecular testing infrastructure for detection of monkeypoxvirus. medRxiv. 2022:2022.06.05.22276011.

16. Nörz D, Tang HT, Emmerich P, Giersch K, Fischer N, Schmiedel S, et al. Rapid Adaptation of Established High-Throughput Molecular Testing Infrastructure for Monkeypox Virus Detection. Emerging Infectious Disease journal. 2022;28(9):1765.

17. Pittman PR, Martin JW, Kingebeni PM, Tamfum J-JM, Mwema G, Wan Q, et al. Clinical characterization and placental pathology of mpox infection in hospitalized patients in the Democratic Republic of the Congo. PLOS Neglected Tropical Diseases. 2023;17(4):e0010384.

18. Pittman PR, Martin JW, Kingebeni PM, Tamfum J-JM, Wan Q, Reynolds MG, et al. Clinical characterization of human monkeypox infections in the Democratic Republic of the Congo. medRxiv. 2022:2022.05.26.22273379.

19. Thy M, Peiffer-Smadja N, Mailhe M, Kramer L, Ferré VM, Houhou N, et al. Breakthrough Infections after Postexposure Vaccination against Mpox. New England Journal of Medicine. 2022;387(26):2477-9.

20. Thy M, Peiffer-Smadja N, Mailhe M, Kramer L, Ferré VM, Houhou-Fidouh N, et al. Breakthrough infections after post-exposure vaccination against Monkeypox. medRxiv; 2022.

21. Yeh T-Y, Contreras G. Recombination shapes 2022 monkeypox outbreak. medRxiv; 2022.

22. Yeh T-Y, Hsieh Z-Y, Feehley MC, Feehley PJ, Contreras GP, Su Y-C, et al. Recombination shapes the 2022 monkeypox (mpox) outbreak. Med. 2022;3(12):824-6.

23. Zucker R, Lavie G, Sagy YW, Arieh NG, Markovits H, Abu-Ahmad W, et al. Risk Assessment of Human Monkeypox Infections for Vaccine Prioritization. Research Square; 2022.

24. Zucker R, Lavie G, Sagy YW, Arieh NG, Markovits H, Ahmad WA, et al. PMC10127933; Risk assessment of human mpox infections: Retrospective cohort study. Clin Microbiol Infect. 2023;20230425.

25. Brand SPC, Cavallaro M, Cumming F, Turner C, Florence I, Blomquist P, et al. The role of vaccination and public awareness in forecasts of Mpox incidence in the United Kingdom. Nat Commun. 2023;14(1):4100.

26. Brand SPC, Cavallaro M, Hilton J, Guzman-Rincon L, House T, Keeling MJ, Nokes DJ. The role of vaccination and public awareness in medium-term forecasts of monkeypox incidence in the United Kingdom. medRxiv; 2022.

27. Sun Y-Q, Chen J-J, Liu M-C, Zhang Y-Y, Wang T, Che T-L, et al. Mapping Global Zoonotic Niche and Interregional Transmission Risk of Monkeypox: A Retrospective Observational Study. SSRN - Lancet prepublication. 2022.

28. Sun YQ, Chen JJ, Liu MC, Zhang YY, Wang T, Che TL, et al. Mapping global zoonotic niche and interregional transmission risk of monkeypox: a retrospective observational study. Global Health. 2023;19(1):58.

29. Frenois-Veyrat G, Gallardo F, Gorgé O, Marcheteau E, Ferraris O, Baidaliuk A, et al. Tecovirimat is highly efficient on the Monkeypox virus lineage responsible for the international 2022 outbreak. 2022.

30. Frenois-Veyrat G, Gallardo F, Gorgé O, Marcheteau E, Ferraris O, Baidaliuk A, et al. Tecovirimat is effective against human monkeypox virus in vitro at nanomolar concentrations. Nature Microbiology. 2022;7(12):1951-5.

31. Gomes JP, Isidro J, Borges V. Multi-country outbreak of monkeypox virus: phylogenomic characterization and signs of microevolution. Research Square prepub. 2022.

32. Isidro J, Borges V, Pinto M, Sobral D, Santos JD, Nunes A, et al. Phylogenomic characterization and signs of microevolution in the 2022 multi-country outbreak of monkeypox virus. Nature Medicine. 2022;28(8):1569-72.

33. Noe S, Zange S, Seilmaier M. Clinical and virological features of first human Monkeypox cases in Germany. Research Square prepub. 2022.

34. Noe S, Zange S, Seilmaier M, Antwerpen MH, Fenzl T, Schneider J, et al. Clinical and virological features of first human monkeypox cases in Germany. Infection. 2023;51(1):265-70.

35. Wang Z, Tober-Lau P, Farztdinov V, Lemke O, Schwecke T, Steinbrecher S, et al. The human host response to monkeypox infection: a proteomic case series study. medRxiv; 2022.

36. Wang Z, Tober‐Lau P, Farztdinov V, Lemke O, Schwecke T, Steinbrecher S, et al. The human host response to monkeypox infection: a proteomic case series study. EMBO Molecular Medicine. 2022;14(11):e16643.

37. Yinka-Ogunleye A. Monkeypox Risk and Mortality Associated with HIV Infection: A National Case Control Study in Nigeria. SSRN - Lancet prepublication. 2022.

38. Yinka-Ogunleye A, Dalhat M, Akinpelu A, Aruna O, Garba F, Ahmad A, et al. Mpox (monkeypox) risk and mortality associated with HIV infection: a national case-control study in Nigeria. BMJ Glob Health. 2023;8(11).

39. Akazawa D, Ohashi H, Hishiki T, Morita T, Iwanami S, Kim KS, et al. Potential anti-monkeypox virus activity of atovaquone, mefloquine, and molnupiravir, and their potential use as treatments. bioRxiv; 2022.

40. Akazawa D, Ohashi H, Hishiki T, Morita T, Iwanami S, Kim KS, et al. Potential Anti-Mpox Virus Activity of Atovaquone, Mefloquine, and Molnupiravir, and Their Potential Use as Treatments. The Journal of Infectious Diseases. 2023;228(5):591-603.

41. Atkinson B, Burton C, Pottage T, Thompson K-A, Ngabo D, Crook A, et al. Infection-competent monkeypox virus contamination identified in domestic settings following an imported case of monkeypox into the UK. medRxiv. 2022:2022.06.27.22276202.

42. Atkinson B, Gould S, Spencer A, Onianwa O, Furneaux J, Grieves J, et al. Monkeypox virus contamination in an office-based workplace environment, England 2022. medRxiv; 2022.

43. Du Z, Shao Z, Bai Y, Wang L, Herrera-Diestra J, Fox S, et al. Reproduction number of monkeypox in the early stage of the 2022 multi-country outbreak. medRxiv; 2022.

44. Du Z, Shao Z, Bai Y, Wang L, Herrera-Diestra JL, Fox SJ, et al. Reproduction number of monkeypox in the early stage of the 2022 multi-country outbreak. Journal of Travel Medicine. 2022;29(8).

45. Knight J, Tan DHS, Mishra S. Maximizing the impact of limited vaccine supply under different epidemic conditions: a two-city monkeypox modelling analysis. medRxiv; 2022.

46. Knight J, Tan DHS, Mishra S. Maximizing the impact of limited vaccine supply under different early epidemic conditions: a 2-city modelling analysis of monkeypox virus transmission among men who have sex with men. Canadian Medical Association Journal. 2022;194(46):E1560-E7.

47. Minasov G, Inniss N, Shuvalova L, Anderson W, Satchell K. Structure of the Monkeypox profilin-like protein A42R reveals potential function differences from cellular profilins. bioRxiv; 2022.

48. Minasov G, Inniss NL, Shuvalova L, Anderson WF, Satchell KJF. Structure of the Monkeypox virus profilin-like protein A42R reveals potential functional differences from cellular profilins. Acta Crystallogr F Struct Biol Commun. 2022;78(Pt 10):371-7.

49. Wurtzer S, Levert M, Dhenain E, Boni M, Tournier JN, Londinsky N, et al. First Detection of Monkeypox Virus Genome in Sewersheds in France: The Potential of Wastewater-Based Epidemiology for Monitoring Emerging Disease. Environmental Science & Technology Letters. 2022;9(11):991-6.

50. Wurtzer S, Levert M, Dhenain E, Boni M, Tournier JN, Londinsky N, et al. First detection of Monkeypox virus genome in sewersheds in France. medRxiv; 2022.

51. Giorgi FM, Pozzobon D, Di Meglio A, Mercatelli D. Genomic characterization of the recent monkeypox outbreak. bioRxiv. 2022:2022.06.01.494368.

52. Giorgi FM, Pozzobon D, Di Meglio A, Mercatelli D. Genomic and transcriptomic analysis of the recent Mpox outbreak. Vaccine. 2024;42(7):1841-9.

53. Betti M, Farrell L, Heffernan J. A Pair Formation Model with Recovery: Application to Monkeypox. medRxiv; 2022.

54. Betti MI, Farrell L, Heffernan J. A pair formation model with recovery: Application to mpox. Epidemics. 2023;44:100693.

55. Chitwood M, Kwon J, Savinkina A, Walker J, Bilinski A, Gonsalves G. Testing, Tracing, and Vaccination Targets for Containment of the US Monkeypox Outbreak: A Modeling Study. medRxiv; 2022.

56. Chitwood MH, Kwon J, Savinkina A, Walker J, Bilinski A, Gonsalves G. Estimated Testing, Tracing, and Vaccination Targets for Containment of the US Mpox Outbreak. JAMA Network Open. 2023;6(1):e2250984-e.

57. Van Dijck C, Hens N, Kenyon C, Tsoumanis A. The roles of unrecognized monkeypox cases, contact isolation and vaccination in determining epidemic size in Belgium. A modelling study. medRxiv; 2022.

58. Van Dijck C, Hens N, Kenyon C, Tsoumanis A. The Roles of Unrecognized Mpox Cases, Contact Isolation and Vaccination in Determining Epidemic Size in Belgium: A Modeling Study. Clinical Infectious Diseases. 2022;76(3):e1421-e3.

59. Tarín-Vicente EJ, Agud-Dios M, Alemany A, Ubals M, Suñer C, Antón A, et al. Clinical Presentation and Virological Assessment of Confirmed Human Monkeypox Virus Cases in Spain : A Prospective Cohort Study. SSRN - Lancet prepublication. 2022.

60. Tarín-Vicente EJ, Alemany A, Agud-Dios M, Ubals M, Suñer C, Antón A, et al. Clinical presentation and virological assessment of confirmed human monkeypox virus cases in Spain: a prospective observational cohort study. The Lancet. 2022;400(10353):661-9.

61. Gu X, Zhang Y, Jiang W, Wang D, Lu J, Gu G, et al. Protective human anti-poxvirus monoclonal antibodies are generated from rare memory B cells isolated by multicolor antigen tetramers. bioRxiv. 2022:2022.06.04.494786.

62. Gu X, Zhang Y, Jiang W, Wang D, Lu J, Gu G, et al. Protective Human Anti-Poxvirus Monoclonal Antibodies Are Generated from Rare Memory B Cells Isolated by Multicolor Antigen Tetramers. Vaccines. 2022;10(7):1084.

63. Yuan P, Tan Y, Yang L, Aruffo E, Ogden N, Belair J, et al. Modelling vaccination and control strategies of outbreaks of monkeypox at gatherings. medRxiv; 2022.

64. Yuan P, Tan Y, Yang L, Aruffo E, Ogden NH, Belair J, et al. Assessing transmission risks and control strategy for monkeypox as an emerging zoonosis in a metropolitan area. medRxiv. 2022:2022.06.28.22277038.

65. Ko Y, Mendoza R, Mendoza VM, Seo Y, Lee J, Jung E. Estimation of monkeypox spread in a non-endemic country considering contact tracing and self-reporting: a stochastic modeling study. medRxiv; 2022.

66. Ko Y, Mendoza Victoria M, Mendoza R, Seo Y, Lee J, Jung E. Estimation of monkeypox spread in a nonendemic country considering contact tracing and self-reporting: A stochastic modeling study. Journal of Medical Virology. 2023;95(1):e28232.

67. Malik AA, Winters MS, Omer SB. Attitudes of the US general public towards Monkeypox. medRxiv. 2022:2022.06.20.22276527.

68. Winters M, Malik AA, Omer SB. Attitudes towards Monkeypox vaccination and predictors of vaccination intentions among the US general public. PLOS ONE. 2022;17(12):e0278622.

69. Wu F, Oghuan J, Gitter A, Mena K, Brown E. Wide mismatches in the sequences of primers and probes for Monkeypox virus diagnostic assays. medRxiv; 2022.

70. Wu F, Oghuan J, Gitter A, Mena KD, Brown EL. Wide mismatches in the sequences of primers and probes for monkeypox virus diagnostic assays. Journal of Medical Virology. 2023;95(1):e28395.

71. Gould S, Atkinson B, Onianwa O, Spencer A, Furneaux J, Grieves J, et al. Air and surface sampling for monkeypox virus in a UK hospital: an observational study. The Lancet Microbe. 2022;3(12):e904-e11.

72. Gould S, Atkinson B, Onianwa O, Spencer A, Furneaux J, Grieves J, et al. Air and surface sampling for monkeypox virus in UK hospitals. medRxiv; 2022.

73. Endo A, Murayama H, Abbott S, Ratnayake R, Pearson CAB, Edmunds WJ, et al. Heavy-tailed sexual contact networks and the epidemiology of monkeypox outbreak in non-endemic regions, May 2022. medRxiv. 2022:2022.06.13.22276353.

74. Endo A, Murayama H, Abbott S, Ratnayake R, Pearson CAB, Edmunds WJ, et al. Heavy-tailed sexual contact networks and monkeypox epidemiology in the global outbreak, 2022. Science. 2022;378(6615):90-4.

75. Schrarstzhaupt IN, Fontes-Dutra M, Diaz-Quijano F. Early estimates of the incidence trend and the reproductive number of the monkeypox epidemic in Brazil. medRxiv; 2022.

76. Schrarstzhaupt IN, Fontes-Dutra M, Diaz-Quijano FA. Early estimates of the incidence trend and the reproductive number of the monkeypox epidemic in Brazil. Travel Medicine and Infectious Disease. 2022;50:102484.

77. Wang H, Abreu de Paulo KJI, Gulzow T, Zimmermann HML, Jonas K. Brief report: Determinants of potential sexual activity reduction in the face of the Monkeypox epidemic. medRxiv; 2022.

78. Wang H, de Paulo K, G├╝ltzow T, Zimmermann HML, Jonas KJ. Brief Report: Determinants of Potential Sexual Activity Reduction in the Face of the Mpox Epidemic. Int J Behav Med. 2024;20240117.
